# Comparison of Postoperative Pain after Root Canal Preparation with Two Reciprocating and Rotary Single-File Systems: A Randomized Clinical Trial

**DOI:** 10.22037/iej.2017.03

**Published:** 2017

**Authors:** Narges Farhad Mollashahi, Eshagh Ali Saberi, Seyed Rohollah Havaei, Mohammad Sabeti

**Affiliations:** a *Oral and Dental Diseases Research Center, Department of Endodontics, Dental School, Zahedan University of Medical Sciences, Zahedan, Iran; *; b *Director, Advanced Specialty Education Program in Endodontics, University of California at San Francisco, USA*

**Keywords:** Endodontic Treatment, Nickel-Titanium Instruments, Postoperative Pain

## Abstract

**Introduction:**

Root canal preparation techniques may cause postoperative pain. The aim of the present study was to compare the intensity of postoperative pain after endodontic treatment using hand files, single file rotary (OneShape), and single file reciprocating (Reciproc) systems.

**Methods and Materials:**

In this single-blind, parallel-grouped randomized clinical trial a total of 150 healthy patients aged between 20 to 50 years old were diagnosed with symptomatic irreversible pulpitis of one maxillary or mandibular molars. The teeth were randomly assigned to three groups according to the root canal instrumentation technique: hand files (control), OneShape and Reciproc. Treatment was performed in a single visit by an endodontist. The severity of the postoperative pain was assessed by the visual analogue scale (VAS) after 6, 12, 24, 48 and 72 h. Data were analyzed using the Kruskal-Wallis and Mann-Whitney U tests.

**Results:**

The patients in control group reported significantly higher mean postoperative pain intensity at 12, 24, 48, and 72 h compared to the patients in the two other groups (*P*<0.05). There was no significant difference in mean intensity of postoperative pain between Reciproc and OneShape at 5 time points (*P*>0.05).

**Conclusion:**

The instrumentation kinematics (single-file reciprocating or single-file rotary) had no impact on intensity of postoperative pain.

## Introduction

Post-endodontic pain is an annoying experience for the patient, undermining the patient-clinician relationship [1]. Despite major improvements in armamentarium and pharmacologic interventions, pain after endodontic treatment remains to be a major problem [2-5] with a frequency ranging from 1.9 to 48% in the literature [1]. This broad range is probably due to differences in study design and the definition of post-operative pain [6]. 

Even when the highest standards are followed, post-endodontic pain of mild (with a frequency of 10-30%) [7, 8] and severe (with a frequency of 6-12%) [8, 9] intensities have been reported in the literature.

Several etiologic factors are attributed to post-operative pain including a history of preoperative pain, defective canal debridement, hyper occlusion, periapical disease and extrusion of debris into the periapical tissue [10]. Extrusion of infected dentin into the periapical tissue has been suggested as a major source of pain after endodontic treatment [11, 12]. Although debris extrusion is an inevitable finding even when instrumentation is limited to the confines of the canal [13], different armamentarium seem to be associated with different amounts of debris extrusion [14-17] with some studies reporting higher amounts of extruded debris after using hand files compared to engine-driven files due to Archamedes screw effect of full rotational movement [18, 19]. Bürklein *et al.* [16] demonstrated that a single file reciprocating system (Reciproc) produced more debris extrusion than two single file rotary systems (OneShape and F360).

Reciproc is a single-file NiTi system, which claims to achieve cleaning and shaping with only one file. In this system reciprocating motion is used in three files (R25-25/0.08, R40-40/0.06 and R50-50/0.05) that should be installed on an electronic motor (VDW Silver Reciproc, VDW Munich, Germany). The files are made of M-wire alloy, which provide higher flexibility and higher resistance to cyclic fatigue, compared to conventional NiTi rotary files [20-22]. OneShape (Micro Méga, Besançon, France) is another single-file NiTi system that uses full-sequence rotary motion that are available in three sizes 25/0.06, 30/0.06 and 40/0.06. The file has variable cross-sections and longer pitch. These properties cause reduction of the preparation time, efficient cleaning, decrease in the bacterial charge similar to that of traditional instruments and lower quantity of apically extruded debris [23]. It has variable cross sections and uses an orifice shaper (EndoFlare) to eliminate the occlusal constraints [16].

While several *in vitro* studies have assessed the debris extrusion in different systems [14-17], few have focused on the clinical outcome [24-27]. Therefore, the purpose of this randomized single-blind study was to compare intensity of postoperative pain after the root canal preparation of molars diagnosed with symptomatic irreversible pulpitis using three different instrumentation techniques: hand, single-file rotary (OneShape), and single-file reciprocating (Reciproc) systems.

## Materials and Methods

This was an active-controlled, single-blinded, parallel-grouped, randomized clinical trial that was carried out under the approval of the Ethics Committee of Zahedan University of Medical Sciences (Grant No.: 7198) and was registered online (ClinicalTrial.gov identification No.: NCT02621034). Assuming *α*=0.05 and a power of 80% (*β*=0.02) the sample size was calculated to be 50 in each group.

Healthy patients aging between 20 to 50 years old with a pulpal status of symptomatic irreversible pulpitis for one maxillary or mandibular molar were treated in the Endodontic Department of Zahedan Dental School. A written consent was obtained before recruitment. 

Exclusion criteria comprised of previous endodontic treatment, a history of medicine intake including corticosteroids, opioids and nonsteroidal anti-inflammatory drugs (NSAIDs) in the past 12 h, pregnancy, complicated anatomy (curves greater than 25 degrees), calcifications, internal and external resorption, open apices, periodontal disease, swelling and abscess, presence of periapical lesions, sensitivity to percussion and lack of occlusal contact. Prior to treatment a list of information regarding age, gender, type of tooth, pulpal and periapical status, presence of periapical lesion and a history of previous treatment were gathered from each patient. 

Allocation was done by a second person and thus the clinician performing the root canal treatment and the patient were blinded. Clinical diagnosis of pulpal status was confirmed by a positive response on cold testing with ENDO-ICE frozen gas (Coltene/Whaledent, Inc., Mahwah, NJ, USA). A visual analogue scale (VAS) was explained to the patients and considering the 0-170 markings on the VAS ruler, the level of pain was scored as follows: *score 0* (mild pain; 0-56), *score 1* (moderate pain; 57-113) and *score 3* (severe pain; 114-170). One clinician assessed the clinical and radiographic information in terms of eligibility and assigned a code to each eligible patient record. The codes were then randomly assigned to three treatment protocols using table of random numbers. The treatment protocol for each patient was placed in a sealed envelope and then the envelope was given to the operator. After working length determination, an endodontist would open the envelope to pick up the treatment procedure; in control group canal instrumentation was conducted using K-files, and in test groups root canal treatment was done with either OneShape )MicroMega, Besancon, France) files or Reciproc (VDW, Munich, Germany) system. A standard initial protocol was carried out in the three groups. 

Teeth were anesthetized using two cartridges of 2% lidocaine with 1:80000 epinephrine (Daroupakhsh, Tehran, Iran) *via *buccal infiltration in the maxilla and inferior alveolar nerve block and long buccal infiltration in the mandibular region. No patient required more than two cartridges of local anesthetic. Isolation was made using rubber dam and access cavity was prepared using round carbide and diamond cylindrical burs in a high-speed hand piece. Upon exposure of the pulp chamber, if hemorrhage was not noticed (indicating pulp necrosis), patients were excluded. The lengths of the canals were determined using an electronic apex locator (Root ZX; J. Morita, Tokyo, Japan) and then confirmed radiographically. The instrumentation sequence used during the treatments in each group followed the procedure recommended by the manufacturer.

Group 1: OneShape files (25/0.06) were used at the manufactures recommended speed (350-450 rpm and 2.5 N/ cm torque) in pecking motion till reaching working length. For wide canals, OneShape Apical (30/0.06, 37/0.06) was used.

Group 2: R25 files (25/0.08) were used in narrow canals, and R40 files (40/0.06) were used in large canals. Three in-and-out motions were applied with stroke lengths not exceeding 3 mm in the cervical, middle, and apical thirds until attaining the established working length.

Group 3 (control group): canals were prepared using stainless steel K-files up to size #25 in smaller and #40 in larger canals. A 0.5 or 1-mm incrementally reducing step-back technique was used to provide tapers of 5% and 10%, respectively depending on the size of the canal.

Following instrumentation with the mentioned techniques the coronal chamber was flushed with 1 mL of 2.5% NaOCl, and agitated ultrasonically for 1 min per canal followed by irrigation with 5 mL 17% EDTA and agitated ultrasonically for 1 min to remove the smear layer. Afterward, irrigation was repeated with 5 mL of 2.5% NaOCl solution and the procedure was completed by irrigation with 5 mL saline solution. The canals were then dried using paper cones and obturated using lateral condensation of gutta percha and AH-26 sealer. The tooth was then temporarily sealed using Cavit (3M ESPE, Seefeld, Germany).

The patients were advised to on-demand use of a 400-mg dosage of Ibuprofen for pain control [24, 28].

At the end of the session VAS questionnaires were handed out to the patients and they were asked to assign a number correlating to their post-treatment pain, with 0 representing no pain and 10 representing the most severe pain imaginable. These scores were marked in intervals of 6, 12, 24, 48 and 72 h following endodontic treatment.

A separate analyzer, unaware of the treatment groups, made phone calls at the designated intervals and recorded the VAS scorings. The data were analyzed using the Kruskal-Wallis and Mann-Whitney U tests. A cut off point of 0.05 was set as the statistical significance.

## Results

A total of 160 patients were included in this study. At the end 10 patients were excluded due to procedural errors; including overfilled canals; and fractured instruments or failure to return the VAS forms and statistical analysis was performed on the remaining 150 participants.

Table 1 summarizes the general characteristics and demographic data of the study groups. There were no differences in age, gender and type of teeth between the groups (*P*>0.05).

The results showed that the intensity of patient’s pain had significantly decreased by 72 h in all groups (*P*<0.05). The highest postoperative pain intensity was recorded in the early stage after the root canal treatment.

The Kruskal-Wallis test showed that the patients in control group reported significantly higher mean postoperative pain intensity at 6, 12, 24, 48, and 72 h compared with the patients in the two other groups (*P*<0.05). There was no significant difference in mean postoperative pain intensity between Reciproc and OneShape at the 5 assessed time points (*P*>0.05) (Table 2).

## Discussion

The aim of this study was to compare the intensity of postoperative pain using two single-file rotary and reciprocation systems (Reciproc and OneShape files).

Due to the subjective and multifactorial nature of pain, many difficulties may arise in both measuring the postoperative level of pain and in controlling the various confounding factors involved. In the present study, the VAS questionnaire was used to quantify pain and categorize it into mild, moderate and severe groups.

By way of randomization, applying a rather large sample size and also implementing strict inclusion criteria the various confounding factors such as age, gender, preoperative pain, type of tooth, pulpal and periapical status, number of treatment visits [7, 29] were kept in similar distribution so that only the shaping technique would remain as the key and distinguishing factor. All attempts were made so that the various procedural steps including the number of anesthetic cartridges used, working length determination, irrigation and obturation procedures would remain identical between the groups. Since different teeth in the same patient would not behave independently only one tooth from each patient was included in the study. Comparison of post-endodontic pain following various instrumentation techniques and armamentarium has been the focus of attention in the recent years. 

**Table 1:** General characteristics and demographic data of patients (*n*=50)

**Demographic and clinical data**	**Reciproc **	**OneShape **	**Control **
**Male**	19	21	23
**Female**	31	29	27
**Mean (SD) of age **	33.2 (5.3)	30.2 (4.28)	31.7 (5.71)
**Max. Molar**	21	20	25
**Man. Molar**	29	30	25

**Table 2:** Mean (SD) of pain intensity in study groups during the first 72 h after treatment

	**Reciproc**	**OneShape**	**Control**	***P*** **-value** *
**Pretreatment**	6.66 (2.12)	5.32 (2.81)	6.36 (3.06)	.074
**After 6 h**	3.22 (3.14)	3.5 (2.91)	5 (2.78)	.006
**After 12 h**	1.52 (2.53)	1.7 (2.17)	3.88 (2.60)	<.001
**After 24 h**	0.9 (2.03)	0.82 (1.52)	2.76 (2.29)	<.001
**After 48 h**	0.5 (1.96)	0.66 (1.37)	1.52 (1.98)	<.001
**After 72 h**	0.46 (1.88)	0.34 (1.09)	0.72 (1.40)	<.001
***P*** **-value**	<.001	<.001	<.001	

* *P-value* *: Kruskal Wallis Test;* *P-value* *: Friedman Test*

The results of this study found no significant difference in post-operative pain between OneShape and Reciproc groups; however, the control group exhibited significantly higher pain intensity compared with the patients in the two other groups. This finding may be attributed to the Archimedes’ screw effect, which minimizes debris extrusion from the apical foramen [30-32]. These results are in line with previous studies that found lower postoperative pain using NiTi rotary files compared to stainless steel hand files, however these studies used different engine-driven instruments [28, 33].Contrary to our findings, previous studies found no significant difference in postoperative pain between stainless steel hand files and NiTi rotary files; this might be due to the use of different rotary systems in their experiment [34, 35]. 

It is well established that extrusion of debris into the periapical region may irritate the periradicular tissues and cause inflammation leading to postoperative pain and flare-ups [11, 36]. While some studies have applied full-sequence rotary files with higher debris extrusion compared to reciprocating rotary files [13, 37], others have reported reciprocating rotary files with more debris extrusion [14, 15, 38]. The variation observed could be attributed to differences in the cross-section, cutting-edge design, taper, tip type, configuration, flexibility, alloy type, number of used files, kinematics, or cutting efficacy [38]. 

Our study, however, has found no significant difference in terms of postoperative pain between reciprocating (Reciproc) and full-sequence rotary files (OneShape). In a randomized multicenter clinical study, Neelakantan *et al.* [24] reported that intensity and duration of postoperative pain was significantly lower in patients undergoing canal instrumentation with Reciproc compared with OneShape. There is a contrast between results of that study and those of the present study, which might be attributed to differences in sample size ( 624 *vs*. 50 in each group), periapical condition (symptomatic apical periodontitis *vs.* normal pulps), preoperative pain categorize on the VAS (severe *vs* moderate), type of teeth (mandibular molars *vs.* mandibular and maxillary molars), number of teeth requiring root canal (two molar in different arch which were treated the same day with a minimum time interval 4 h *vs.* one molar), sealer and obturation technique (MTA plus- warm vertical condensation *vs*, AH-26- lateral condensation), Micro-computed tomography (µCT) studies have shown that reciprocating motion provides better shaping, with less incidence of canal transportation, compared to rotary files [39]. OneShape files have shown significantly higher canal straightening and apical transportation compared to Reciproc [40]. This could be one of the reasons for increased intensity of postoperative pain by OneShape. It should be noted that the results of only one clinical study cannot be generalized to all clinical cases, and more studies regarding this matter are required; therefore, more studies, with larger sample sizes are warranted to further investigate the drawbacks and benefits of these two systems with regards to pain after endodontic treatment.

## Conclusion

This study found significantly higher levels of post-operative pain in the control group using K-files compared to Reciproc and OneShape groups. No significant difference was found between Reciproc and OneShape groups in terms of pain after endodontic treatment. It seems that the instrumentation kinematics had no impact on intensity of postoperative pain.
